# Medical Gas Plasma Promotes Apoptosis Accompanied by Immunomodulation in Gastrointestinal Tumor Cells

**DOI:** 10.3390/cancers18213526

**Published:** 2026-11-03

**Authors:** Lisa-Marie Zietz, Tino Pritzkow, Debora Singer, Alice Martinet, Lea Miebach, Sander Bekeschus

**Affiliations:** 1 ZIK *plasmatis*, Leibniz Institute for Plasma Science and Technology (INP), Felix-Hausdorff-Str. 2, 17489 Greifswald, Germany; 2 Department of Dermatology, Venerology, and Allergology, Rostock University Medical Center, Strempelstr. 13, 18057 Rostock, Germany; 3 Department of Hematology and Oncology, Greifswald University Medical Center, Sauerbruchstr., 17475 Greifswald, Germany

**Keywords:** colorectal cancer, cholangiocellular carcinoma, kINPen, reactive oxygen species, RONS

## Abstract

Background/Objectives: Medical plasma is a promising therapeutic approach in oncology due to its ability to generate reactive oxygen and nitrogen species (RONS) and modulate tumor cell viability and phenotype. Therefore, medical plasma could expand the treatment options for highly prevalent or difficult-to-treat cancer entities such as gastrointestinal cancers. Methods: This study investigated the effects of medical plasma generated by the clinically certified argon plasma jet kINPen on two gastrointestinal tumor cell lines (HT-29, MUG-CCArly) with a focus on cytotoxicity, immunomodulation, and redox-dependent mechanisms. Results: Plasma produced a mixture of reactive species under the applied experimental conditions and was able to reduce metabolic activity and cell viability via a redox-dependent induction of apoptosis in both cell lines. Plasma exposure altered the expression of cell-surface markers with potentially immunostimulatory roles such as CD40, CRT, and HMGB1, as well as more immunoregulatory markers like CD47 and PD-L1 (CD274). Additionally, modulated secretion profiles marked by increased pro-inflammatory molecules such as IL-8 and MIP-3a (CCL20) were detected. Conclusion: Overall, congruent but also cell line-dependent responses to plasma treatment were observed that featured a modified immunological phenotype of tumor cells with potentially immunostimulatory but also immunomodulatory aspects, highlighting the potential of medical gas plasma technology as a multimodal therapeutic approach in gastrointestinal oncology.

## Simple Summary

The use of a certified medical plasma jet was investigated for its mechanisms of action in killing gastrointestinal cancer cells. A large intestine and a bile duct cancer cell line were used to test the direct toxicity and the impact on immune-relevant molecule production of the plasma treatment. It was detected that the plasma mediated an anticancer effect by inducing programmed cell death and modified the immune surface molecule profile and secretion of messenger molecules in a cell line-specific way. The plasma effects were attributed to a change in cellular oxidation processes since the plasma jet produces oxidative molecules, and a scavenger of these could partly reduce the observed effects. These findings support the possible use of plasma as a tool in gastrointestinal cancer therapy and indicate the need for further investigations on the interplay of direct anticancer effects and immune-mediated processes of medical plasma treatment.

## 1. Introduction

Gastrointestinal (GI) cancers remain among the leading causes of cancer-related morbidity and mortality worldwide and comprise a heterogeneous group of malignancies, including colorectal cancer (CRC) and cholangiocarcinoma (CCA) [1]. While treatment strategies such as surgery, chemotherapy, radiotherapy, targeted therapies, and immunotherapy have improved clinical outcomes, their efficacy is frequently limited by late-stage diagnosis, metastatic dissemination, tumor heterogeneity, and the development of therapy resistance [2,3,4]. Consequently, the development of novel therapeutic approaches capable of selectively targeting tumor cells while simultaneously promoting anti-tumor immune responses has become an important focus of translational cancer research.

Against this background, medical plasma has emerged as a promising approach in experimental cancer research [5,6]. Medical plasma refers to a partially ionized gas generated at near-body temperature that contains a complex mixture of reactive oxygen and nitrogen species (RONS), charged particles, electric fields, and ultraviolet radiation [7,8]. Unlike conventional thermal plasma, cold gas plasma can be applied to biological tissues without causing substantial thermal damage. Among the plasma devices developed for biomedical applications, the argon-driven kINPen represents a clinically approved medical plasma device and has therefore particular translational relevance for cancer research. Its well-characterized and standardized plasma output enables the investigation of plasma-induced biological effects under conditions that are closely related to potential clinical applications [9].

The anti-tumor effects of medical plasma are primarily driven by plasma-generated RONS, which induce oxidative damage to cellular components and modulate signaling pathways, thereby activating cell-death mechanisms in tumor cells. Typical plasma-derived RONS include hydrogen peroxide (H_2_O_2_), superoxide (O_2_^−^), hydroxyl radicals (HO•), nitric oxide (NO), peroxynitrite (ONOO^−^), and other reactive species [10,11,12]. Importantly, cancer cells typically exhibit elevated basal levels of ROS compared with non-malignant cells as a consequence of metabolic reprogramming, genomic instability, mitochondrial dysfunction, and alterations in the tumor microenvironment [13]. This increased oxidative stress makes tumor cells more sensitive to additional RONS generated during plasma exposure, which can lead to oxidative damage and cell death [14,15,16]. Previous studies have demonstrated that medical plasma exerts multiple anti-tumor effects in colorectal cancer models, including inhibition of cell proliferation through cell-cycle arrest, activation of RONS-dependent apoptotic signaling pathways, as well as suppression of migration and invasion, highlighting its therapeutic potential beyond direct cytotoxicity [17,18,19]. However, the relative contribution of plasma-derived RONS to these effects and their applicability across different gastrointestinal malignancies remain incompletely understood.

Importantly, the plasma sources investigated in GI cancer cell lines have been highly heterogeneous. In colorectal cancer, studies using the kINPen have predominantly employed murine CT26 cells, either through direct plasma exposure or by applying plasma-treated liquids [20,21,22]. In contrast, work using human colorectal cancer cells, including HT-29, has employed plasma sources other than kINPen or plasma-treated liquids rather than direct exposure to plasma [17,18,19,23,24]. In human cholangiocarcinoma models, including EGI-1 and HuCCT-1 cells, studies to date have predominantly relied on experimental plasma sources [25,26,27]. Thus, despite the clinical certification and translational relevance of the kINPen, its direct effects on human gastrointestinal cancer cells, particularly on cholangiocarcinoma models, remain insufficiently characterized.

**Figure 1:**
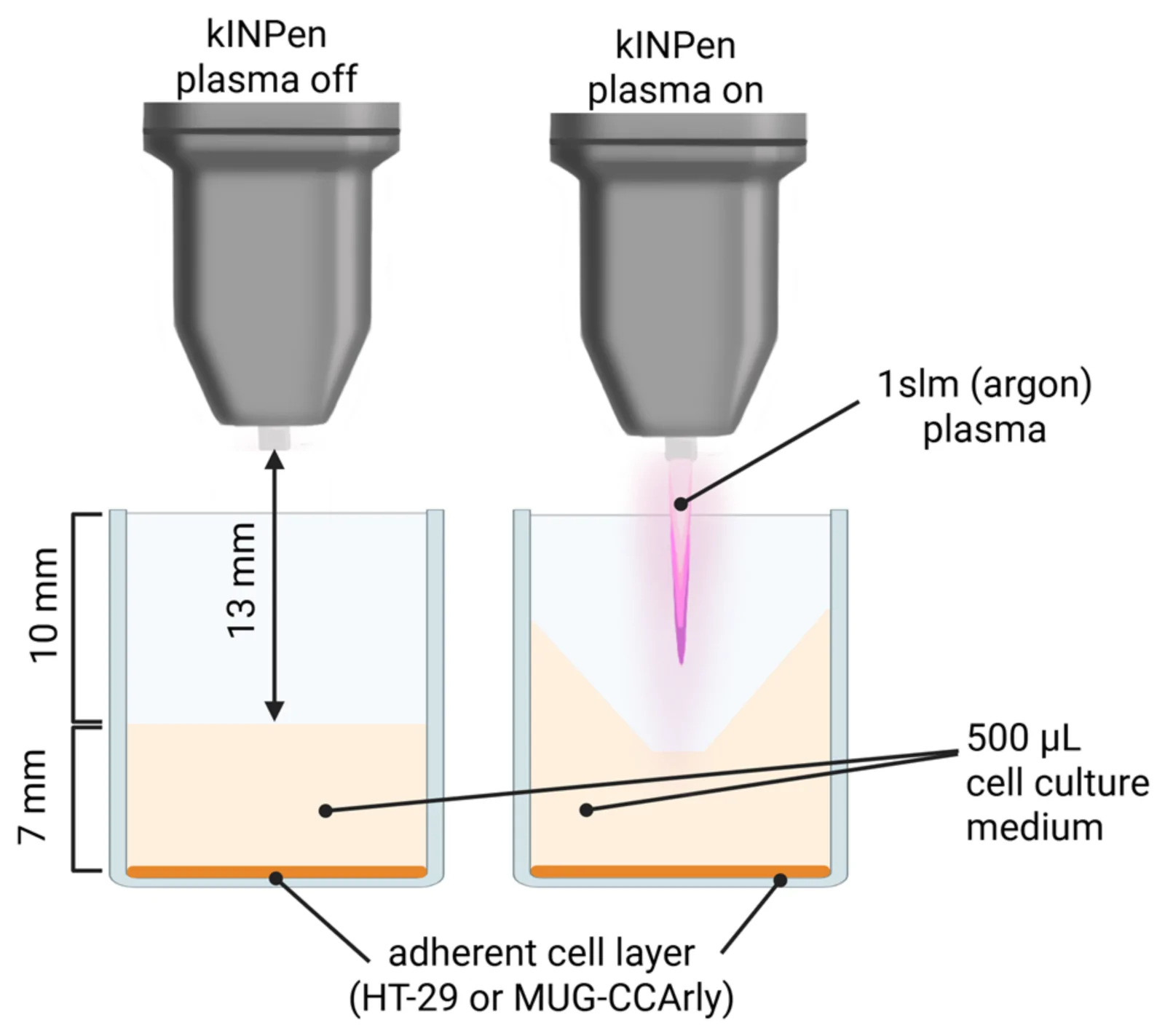
**Plasma treatment configuration.** Schematic of the experimental setup depicting the position of the plasma jet under non-operating (plasma off) and operating (plasma on) conditions.

Therefore, this study investigated the effects of medical plasma generated by the argon plasma jet kINPen in two gastrointestinal cancer cell lines: HT-29 colorectal adenocarcinoma cells and MUG-CCArly bile duct carcinoma cells (Figure 1). The aim was to assess plasma-induced cytotoxicity and immunomodulating effects, and to characterize the underlying RONS-driven mechanisms. By using a clinically certified plasma device, this study addresses an important step toward the translational evaluation of medical plasma in gastrointestinal oncology.

## 2. Materials and Methods

### 2.1. Cell Culture

Two different human cancer cell lines, the colorectal adenocarcinoma cell line HT-29 (ATCC: HTB-38) and the bile duct carcinoma cell line MUG-CCArly (DSMZ: ACC 960), were used in this study. The cells were cultured under standard conditions (37 °C, 5% CO_2,_ and 95% humidity) using their respective culture media: Dulbecco’s Modified Eagle’s Medium (DMEM) for HT-29 cells and DMEM/F12 (1:1) for MUG-CCArly cells (both media from PAN-Biotech, Aidenbach, Germany). The media were supplemented with 10% fetal bovine serum (FBS), 1% L-glutamine, and 1% penicillin/streptomycin (all PAN-Biotech).

### 2.2. Application of Medical Plasma

Cold physical plasma treatment was performed using the atmospheric-pressure plasma jet kINPen (neoplas, Greifswald, Germany) [9], operated with argon gas at a flow rate of one standard liter per minute. For cell culture experiments, 4 × 10^5^ cells were seeded in 500 µL of fully supplemented Roswell Park Memorial Institute (RPMI) 1640 medium in a 48-well plate and left to adhere overnight. The next day, plasma treatment was performed in a near-conductive configuration, with the kINPen ceramic nozzle positioned 13 mm above the cell culture medium. In general, four experimental groups were included: medical plasma treatment, plasma combined with the antioxidant N-acetylcysteine (NAC; 8 mM; added before the treatment), argon gas control (without ignition of the plasma), and untreated controls.

### 2.3. Quantification of Plasma-Generated Reactive Species in Liquid

To characterize RONS generated by the medical plasma, phosphate-buffered saline (PBS; 500 µL per well) in 48-well cell culture plates was exposed to plasma treatment times ranging from 0 to 300 s. The evaporated volume was compensated with ddH_2_O prior to performing the assays for each treatment time. Hydrogen peroxide (H_2_O_2_) concentrations were quantified using the Amplex UltraRed reagent (AUR; Thermo Fisher Scientific, Bremen, Germany), while nitrite (NO_2_^−^) levels were determined using the Measure-IT High-Sensitivity Nitrite Assay Kit (Thermo Fisher Scientific, Germany). In parallel, the formation of short-lived RONS was assessed by treating the fluorescent probes 4,5-diaminofluorescein (DAF-2; 5 µM), aminophenyl fluorescein (APF; 5 µM), and hydroxyphenyl fluorescein (HPF; 5 µM) (all from Enzo Life Sciences, Wiesbaden, Germany) in PBS. All fluorescence measurements were acquired using an Infinite F200 microplate reader (Tecan, Männedorf, Switzerland) at λ_ex_ 485 nm and λ_em_ 535 nm.

### 2.4. IC_25_ Metabolic Activity Assessment

Twenty hours after medical plasma treatment, cell viability was assessed by adding resazurin (Cayman Chemical, Hamburg, Germany) [28] to the cell culture medium to a final concentration of 100 µM, followed by incubation for 4 h at 37 °C. The conversion of resazurin into fluorescent resorufin was measured using a microplate reader (Infinite F200 pro; Tecan, Männedorf, Switzerland) at λ_ex_ 535 nm and λ_em_ 590 nm. Mean fluorescence intensities of treated samples were normalized to untreated controls and log-transformed to determine IC_25_ values by nonlinear regression using a least-squares fitting approach. The IC_25_ value was defined as the plasma treatment duration required to reduce cellular metabolic activity by 25% relative to untreated controls. The resulting treatment duration was then used as the standardized plasma exposure time for all subsequent cell culture experiments.

### 2.5. Detection of Intracellular Oxidation and Cytotoxicity

To assess cellular oxidation, cells were incubated for 30 min at 37 °C prior to medical plasma treatment with 1 µM DAPI (Carl Roth, Karlsruhe, Germany), 1 µM MitoSOX Red (Thermo Fisher Scientific, Germany), and 5 µM CellROX Green (Thermo Fisher Scientific, Germany). Immediately after medical plasma treatment, fluorescence images were acquired using an Operetta CLS high-content imaging system (PerkinElmer, Hamburg, Germany). For each well, 17 fields of view were captured using a 20× air objective. In addition, changes in cellular thiol levels were assessed. Two hours after treatment, cells were harvested and incubated with 5 µM Thioltracker Violet (Thermo Fisher Scientific, Germany) for 20 min. Thioltracker fluorescence was analyzed by flow cytometry using a CytoFLEX flow cytometer (Beckman Coulter, Krefeld, Germany). Plasma-induced cytotoxicity was assessed 24 h after medical plasma treatment using the Operetta CLS high-content imaging system. Cells were incubated with 0.5 µM MitoTracker Deep Red (Thermo Fisher Scientific, Germany), 1 µM CellEvent Caspase reagent (Thermo Fisher Scientific, Germany), and 1 µM DAPI for 30 min at 37 °C prior to image acquisition. Algorithm-based image analysis was performed using Harmony software version 5.2 (PerkinElmer, Germany) to quantify relative fluorescence intensities of MitoTracker, MitoSOX, and CellROX, or cell area, and DAPI^+^ and Caspase 3/7^+^ cells in the oxidation or the cytotoxicity setup, respectively. Statistical analysis was performed using ordinary one-way ANOVA with multiple comparisons versus untreated controls (utr), in addition to paired Wilcoxon matched-pairs signed-rank tests for the comparison between IC_25_ and IC_25_+NAC.

### 2.6. Flow Cytometry Analysis of Surface Marker Expression

Twenty-four hours after plasma treatment, adherent cells were detached, resuspended in PBS, and incubated with fluorochrome-conjugated antibodies (Table 1). Cell viability was determined using iFlour860 (Thermo Fisher Scientific, Germany). Flow cytometric analysis was performed using a CytoFLEX flow cytometer. Data compensation and analysis were carried out using Kaluza Analysis Software (Beckman-Coulter, Germany) with dedicated compensation matrices and analysis protocols for each panel. For each experimental condition, fold changes (FC) were calculated relative to the untreated control. For visualization and statistical analysis, FC values were transformed according to:

$$T(\mathrm{FC})\;=\{\begin{array}{r} 1-1/\mathrm{FC},\;\mathrm{FC}<1\\ \mathrm{FC}-1,\;\mathrm{FC}\geq 1 \end{array}$$

where T denotes the transformed fold change. This transformation centers the untreated control at 0 while preserving symmetry between increases and decreases. Accordingly, a doubling (FC = 2) is represented by +1, whereas a halving (FC = 0.5) is represented by −1.

### 2.7. Cytokine and Chemokine Quantification

The concentrations of secreted factors in cell culture supernatants collected after plasma treatment were determined using a bead-based multiplex immunoassay (LEGENDplex; BioLegend, Amsterdam, The Netherlands) targeting ENA-78, Eotaxin, GROα, IL-8, IP-10, I-TAC, MCP-1, MIG, MIP-1α, MIP-1β, MIP-3α, and TARC. Data acquisition was performed by flow cytometry, where individual bead populations were identified based on their intrinsic fluorescence characteristics, and analyte concentrations were quantified from the corresponding PE fluorescence intensity. Standard curves were generated using the supplied reference standards, and cytokine concentrations were calculated with the dedicated LEGENDplex data analysis software (BioLegend, Amsterdam, The Netherlands). IL-6 concentrations were quantified using an enzyme-linked immunosorbent assay (ELISA) kit (BioLegend, Amsterdam, The Netherlands) according to the manufacturer’s protocol.

### 2.8. Statistical Analysis

For all cell culture experiments, at least three independent biological replicates were conducted. Statistical analyses and data visualization were conducted using Prism software version 9.5.1 (GraphPad Software, Boston, MA, USA). Depending on the experimental design, paired or unpaired Student’s *t*-tests, one-way analysis of variance (ANOVA), or nonlinear regression were applied as appropriate, and statistical significance was defined as *p* < 0.05. Statistical results were reported in accordance with APA (American Psychological Association) guidelines, with significance levels indicated as follows: *p* < 0.05 (*), *p* < 0.01 (**), *p* < 0.001 (***).

## 3. Results

### 3.1. Time-Dependent Generation of RONS in Plasma-Treated PBS

**Figure 2:**
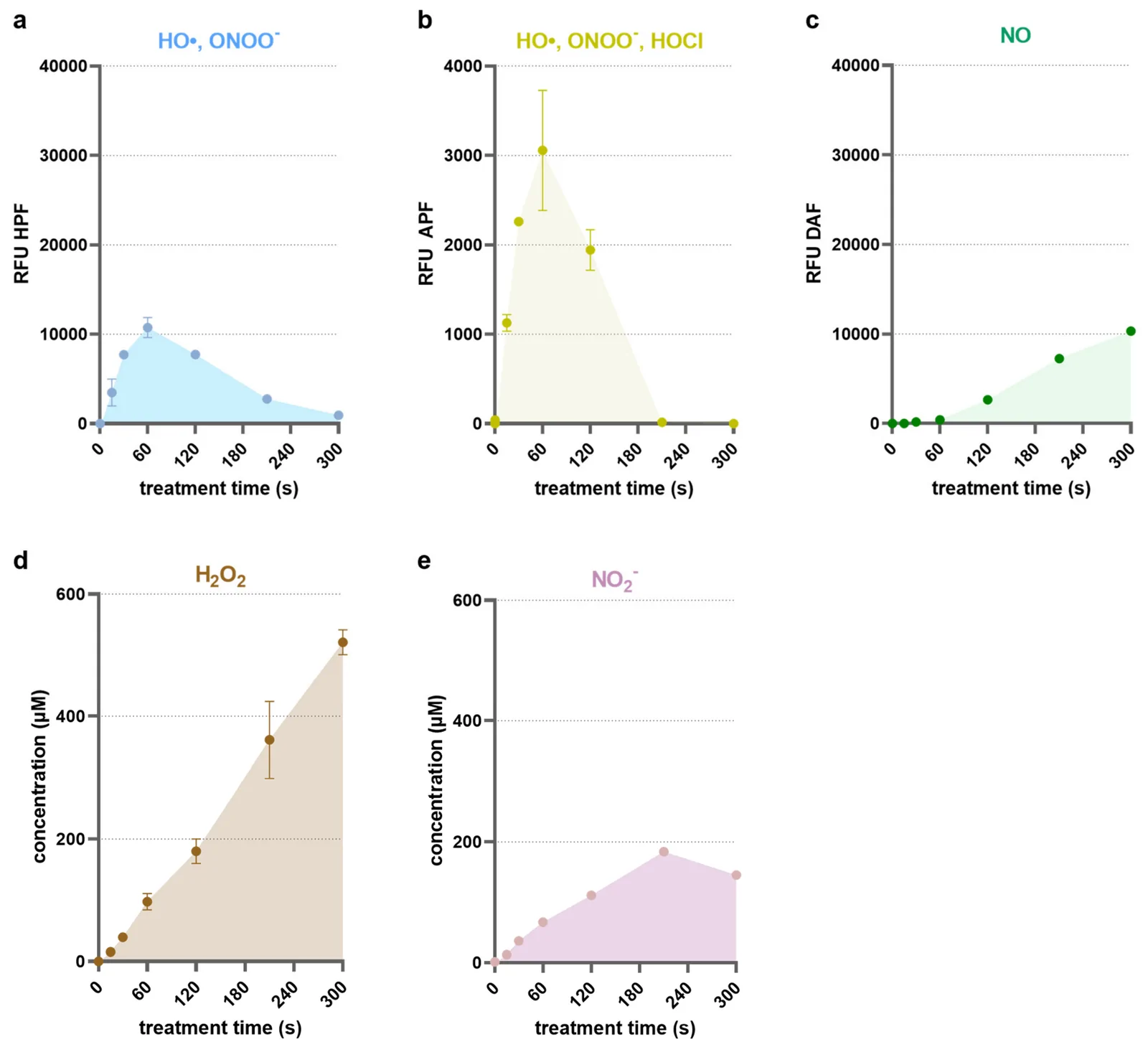
**Time-dependent generation of reactive oxygen and nitrogen species (RONS) in plasma-treated PBS.** Relative fluorescence intensities (RFU) of probes indicative of short-lived species, including (**a**) HPF for detection of hydroxyl radical (HO•) and peroxynitrite (ONOO^−^), (**b**) APF for detection of HO•, ONOO^−^, and hypochlorous acid (HOCl), and (**c**) DAF-2 for detection of nitric oxide (NO); absolute levels of long-lived species, including (**d**) hydrogen peroxide (H_2_O_2_) and (**e**) nitrite (NO_2_^−^). Data are derived from four technical replicates.

Plasma treatment of phosphate-buffered saline (PBS) using the kINPen medical plasma device resulted in a treatment-time-dependent generation of RONS (Figure 2).

Fluorescence-based detection of short-lived reactive species revealed distinct temporal dynamics depending on the probe. Aminophenyl fluorescein (APF) (Figure 2a) and hydroxyphenyl fluorescein (HPF) (Figure 2b), which primarily detect highly reactive species, such as hydroxyl radicals and peroxynitrite, showed a pronounced increase in fluorescence intensity at short plasma exposure times. Both probes reached a maximum signal after 60 s of plasma treatment. Beyond this peak, fluorescence intensity gradually decreased with longer treatment times. In contrast, 4,5-diaminofluorescein (DAF-2), used for the detection of nitric oxide-derived reactive nitrogen species, exhibited a continuous increase in fluorescence over the entire treatment range (Figure 2c). Quantification of long-lived reactive species further confirmed the build-up of stable RONS in PBS. Hydrogen peroxide (H_2_O_2_) concentrations increased continuously with plasma exposure time, reaching 521 µM after 300 s of treatment (Figure 2d). Similarly, nitrite (NO_2_^−^) levels increased in a time-dependent manner, reaching a maximum of 184 µM at 210 s, followed by a slight decrease at 300 s (Figure 2e).

### 3.2. Plasma Exposure Induces Dose-Dependent Cytotoxicity in Gastrointestinal Cancer Cells

**Figure 3:**
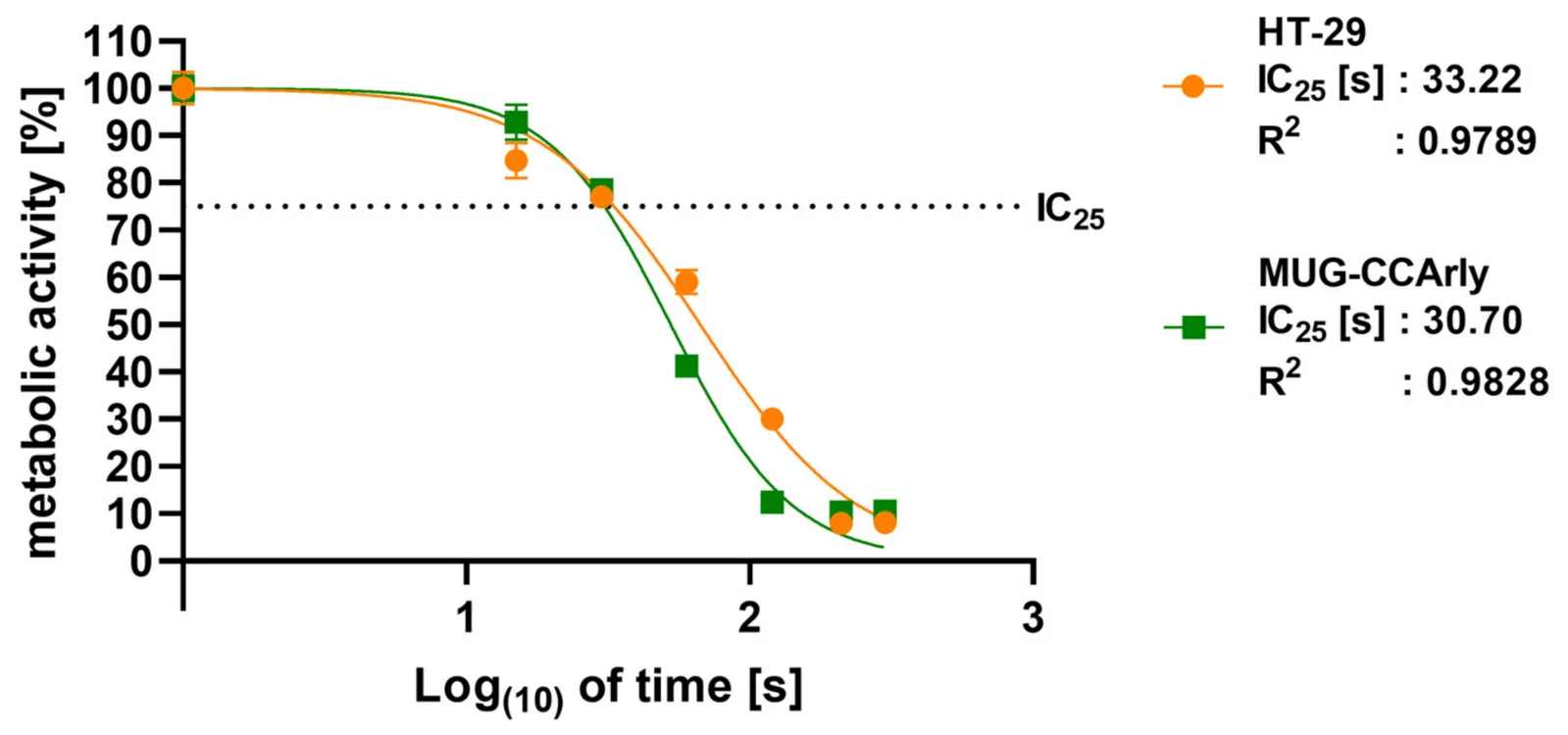
**IC_25_ determination in plasma-treated gastrointestinal cancer cells.** Metabolic activity of HT-29 colon carcinoma and MUG-CCArly cholangiocellular carcinoma cells decreased in a dose-dependent manner following plasma exposure, as assessed by the resazurin assay and normalized to untreated controls. Sigmoidal regression revealed comparable IC_25_ values for both cell lines (~30 s). Data are derived from four technical replicates.

The effect of plasma treatment on cellular metabolic activity was assessed using a resazurin-based assay and calculated relative to the untreated control (Figure 3).

**Figure 4:**
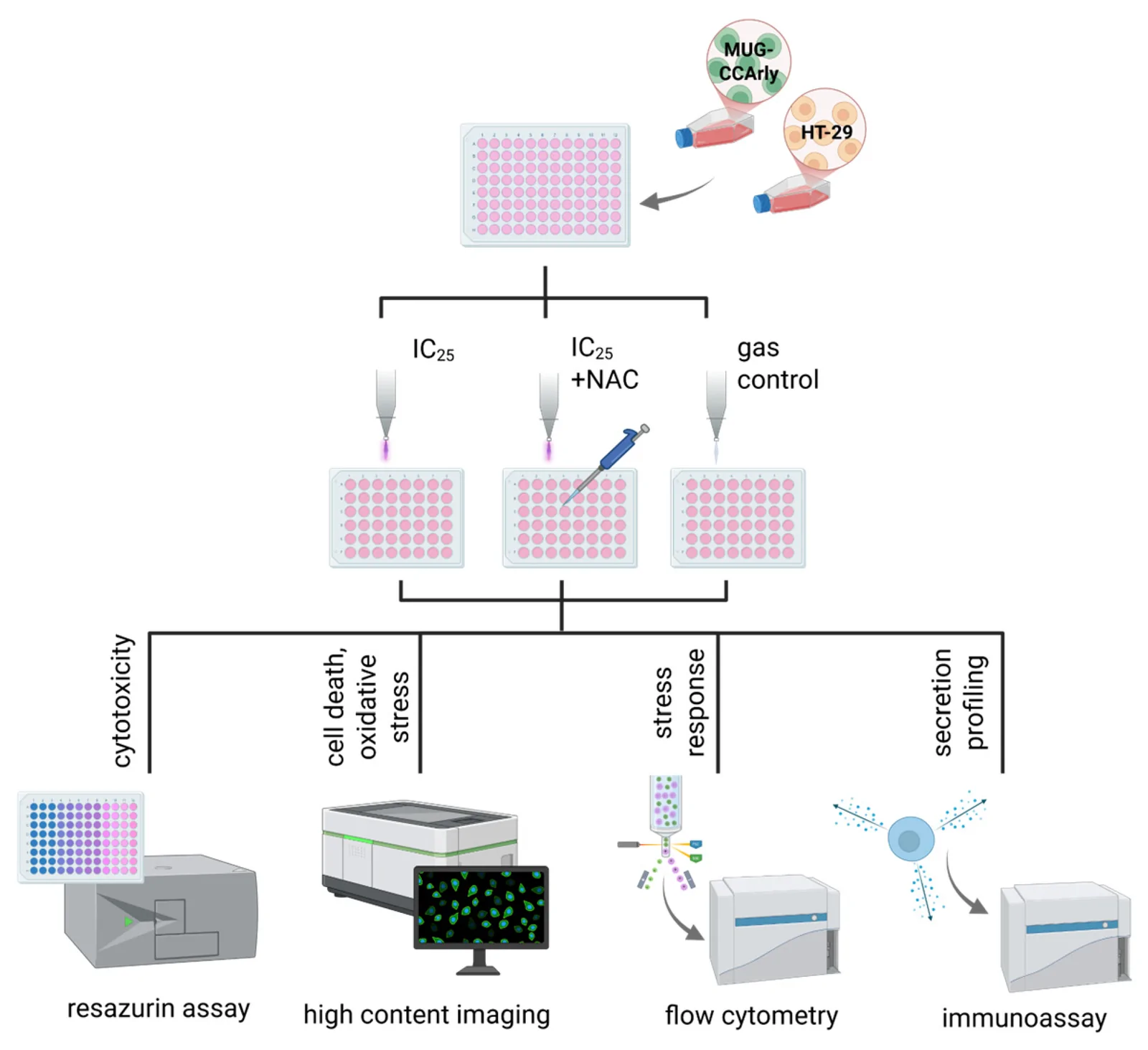
**Study overview.** Two gastrointestinal cancer cell lines (HT-29 and MUG-CCArly) were treated with cold physical plasma to assess cytotoxicity, oxidative stress, cell death, stress response, and secretion profiling using plate reader-, imaging-, and flow cytometry-based assays. The plasma exposure time corresponding to the IC_25_, as previously determined using the resazurin assay, was applied. To investigate the mechanisms underlying the observed treatment effects, the effects of plasma treatment in the presence of N-acetylcysteine (NAC), a RONS scavenger, were assessed alongside untreated and argon gas-only-treated (i.e., plasma = off) controls.

Both HT-29 and MUG-CCArly cells exhibited a clear time-dependent decrease in metabolic activity upon plasma exposure. The dose–response relationships were well described by sigmoidal nonlinear regression fits, as indicated by high coefficients of determination (R^2^). The calculated IC_25_ values were comparable between both cell lines, with HT-29 showing an IC_25_ of 33 s and MUG-CCArly an IC_25_ of 31 s. Both IC_25_ values were in the range of approximately 30 s of plasma treatment, which was consequently used as the standardized treatment duration for all subsequent experiments (Figure 4) in both cell lines.

### 3.3. High-Content Imaging Reveals Plasma-Induced Oxidative Stress and Cell Death

**Figure 5:**
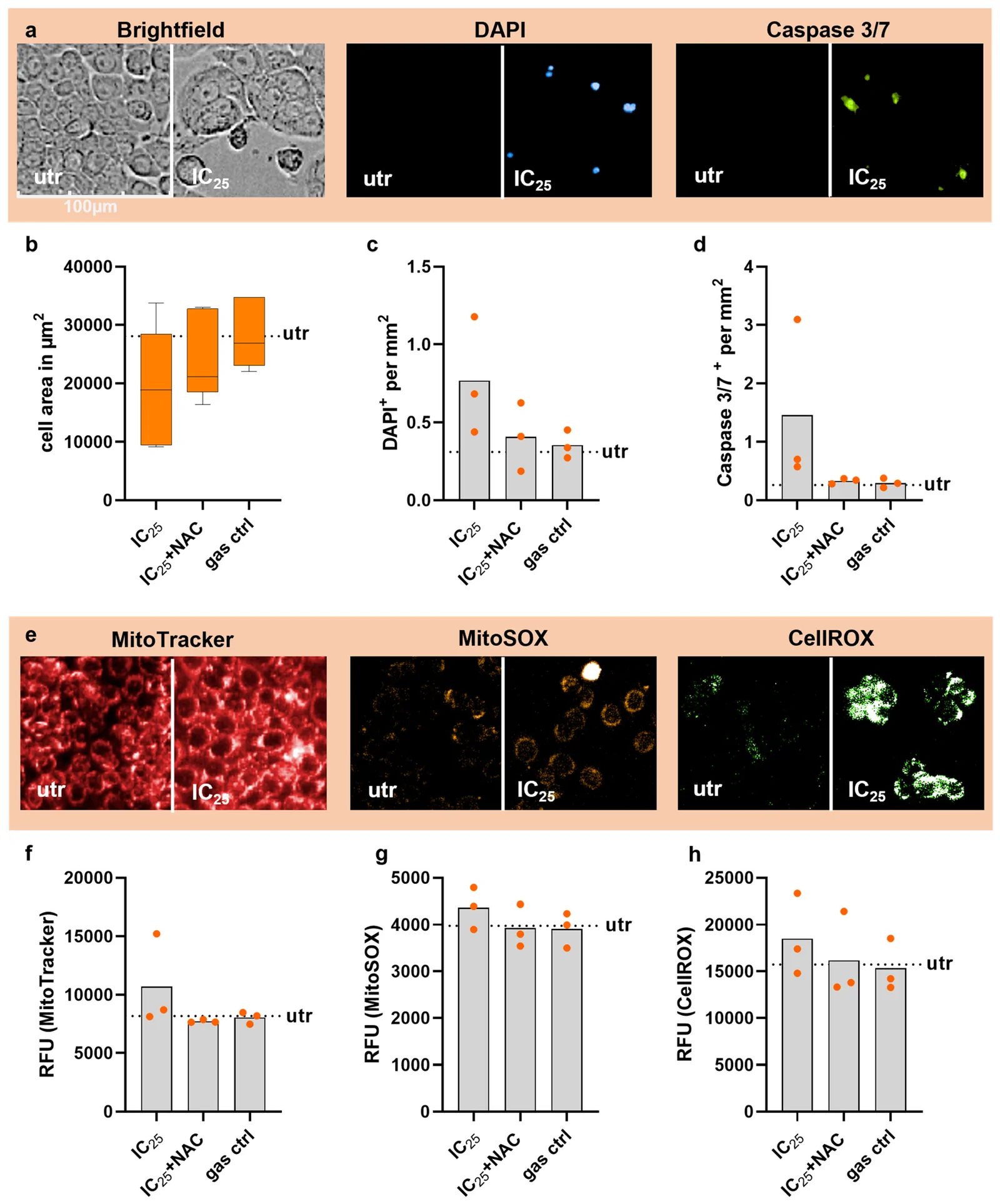
**High-content imaging analysis of HT-29 cells.** Representative images (**a**,**e**) comparing IC_25_-treated cells with untreated cells are shown above the corresponding graph. Quantification of the cytotoxicity-indicating properties (**b**) cell area (µm^2^), (**c**) DAPI-positive (cells/mm^2^), (**d**) Caspase-3/7-positive (cells/mm^2^), and (**f**) MitoTracker fluorescence intensity (RFU) 24 h after treatment; quantification of intracellular oxidation via (**g**) MitoSOX fluorescence intensity (RFU) and (**h**) CellROX fluorescence intensity (RFU) immediately after treatment. Statistical significance relative to the untreated control was determined using ordinary one-way ANOVA followed by Dunnett’s multiple comparisons test and is indicated by asterisks within the corresponding bars. Statistical significance between the IC_25_ and IC_25_+NAC groups was assessed using paired Wilcoxon signed-rank test and is indicated above the corresponding bars. Data are derived from three independent biological replicates, each analyzed in duplicate. The dashed line indicates the untreated control. Only statistically significant differences are marked, whereas all other comparisons were not significant.

**Figure 6:**
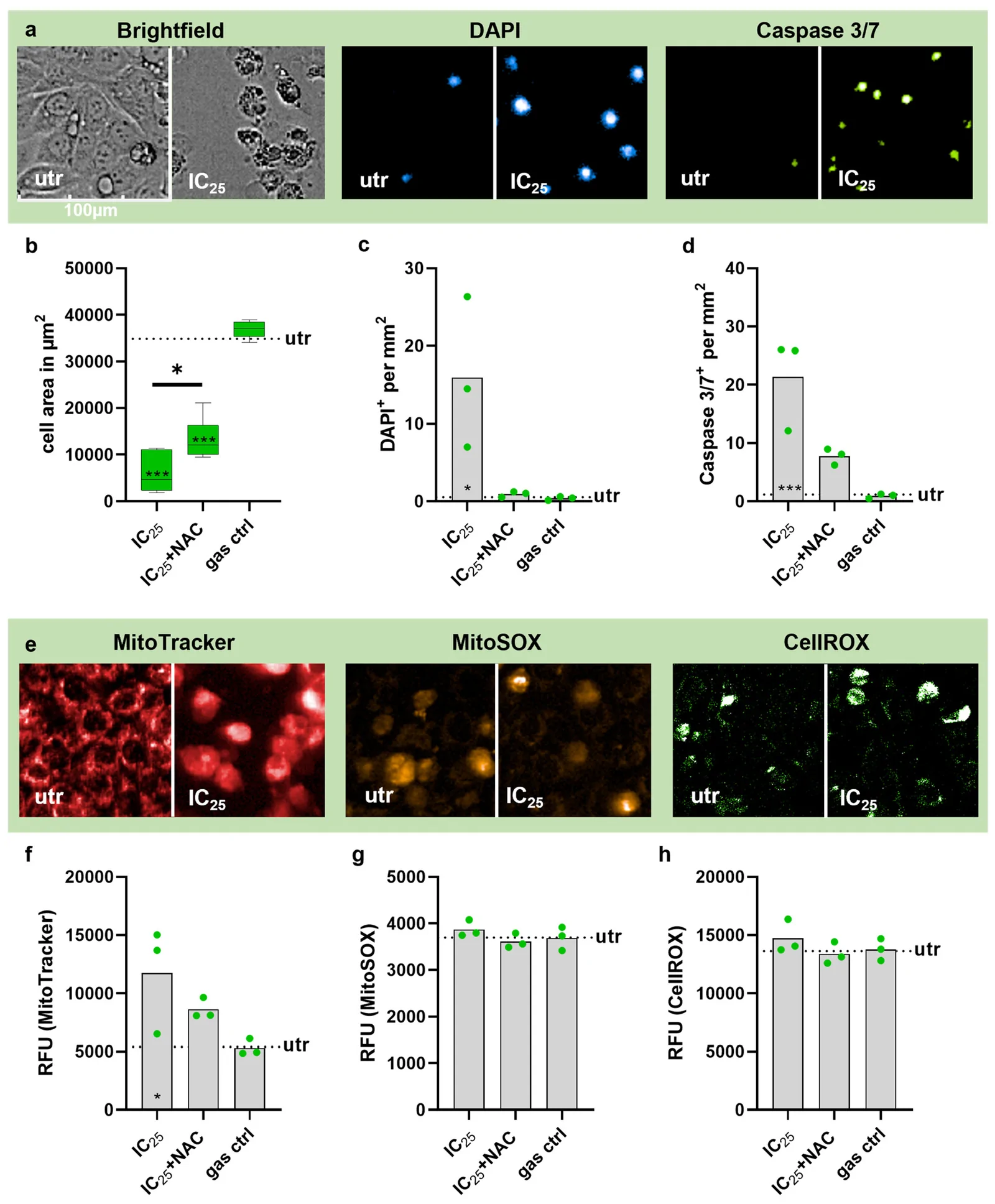
**High-content imaging analysis of MUG-CCArly cells.** Representative images (**a**,**e**) comparing IC_25_-treated cells with untreated cells are shown above the corresponding graph. Quantification of the cytotoxicity-indicating properties (**b**) cell area (µm^2^), (**c**) DAPI-positive (cells/mm^2^), (**d**) Caspase-3/7-positive (cells/mm^2^), and (**f**) MitoTracker fluorescence intensity (RFU) 24 h after plasma treatment; quantification of intracellular oxidation via (**g**) MitoSOX fluorescence intensity (RFU) and (**h**) CellROX fluorescence intensity (RFU) immediately after treatment. Statistical significance relative to the untreated control was determined using ordinary one-way ANOVA followed by Dunnett’s multiple comparisons test and is indicated by asterisks within the corresponding bars (* *p* < 0.05, *** *p* < 0.001). Statistical significance between the IC_25_ and IC_25_+NAC groups was assessed using paired Wilcoxon signed-rank test and is indicated above the corresponding bars. Data are derived from three independent biological replicates, each analyzed in duplicate. The dashed line indicates the untreated control. Only statistically significant differences are marked, whereas all other comparisons were not significant. Green dots indicate independent biological replicates.

To further investigate the biological effects of medical argon plasma treatment at the previously determined IC_25_ exposure time (30 s), immediate intracellular oxidation and cytotoxicity after 24 h were assessed using high-content imaging in both HT-29 (Figure 5) and MUG-CCArly (Figure 6) cells. In HT-29 cells (Figure 5a), medical plasma treatment resulted in a statistically non-significant reduction in the cellular area from 28,000 µm^2^ in untreated cells to 19,500 µm^2^ following IC_25_ treatment (Figure 5b). Pre-treatment with NAC partially attenuated this effect, resulting in a cellular area of 24,000 µm^2^. The density of DAPI-positive cells increased from 0.31 DAPI^+^/mm^2^ in untreated control to 0.77 DAPI^+^/mm^2^ after IC_25_ treatment. In contrast, cells pre-treated with NAC showed intermediate values (Figure 5c). A comparable pattern was detected for caspase-positive cells. IC_25_ treatment increased the number of caspase-positive cells from 0.3 Caspase^+^/mm^2^ in untreated cells to 1.5 Caspase^+^/mm^2^. Pre-treatment with NAC reduced this response to 0.33 Caspase^+^/mm^2^ (Figure 5d). Despite the consistent trend, neither the comparisons to untreated controls nor the paired comparison between IC_25_ and IC_25_+NAC treatment reached statistical significance. MitoTracker fluorescence (Figure 5e) also showed a treatment-dependent increase following IC_25_ exposure. However, this effect was not statistically significant (Figure 5f). MitoSOX and CellROX fluorescence also exhibited only minor treatment-dependent changes, with no significant differences between groups (Figure 5g,h). In MUG-CCArly cells (Figure 6a), the reduction in cellular area was more pronounced, decreasing from 35,000 µm^2^ in untreated cells to 6000 µm^2^ following IC_25_ treatment and 13,000 µm^2^ following IC_25_+NAC treatment.

**Figure A1:**
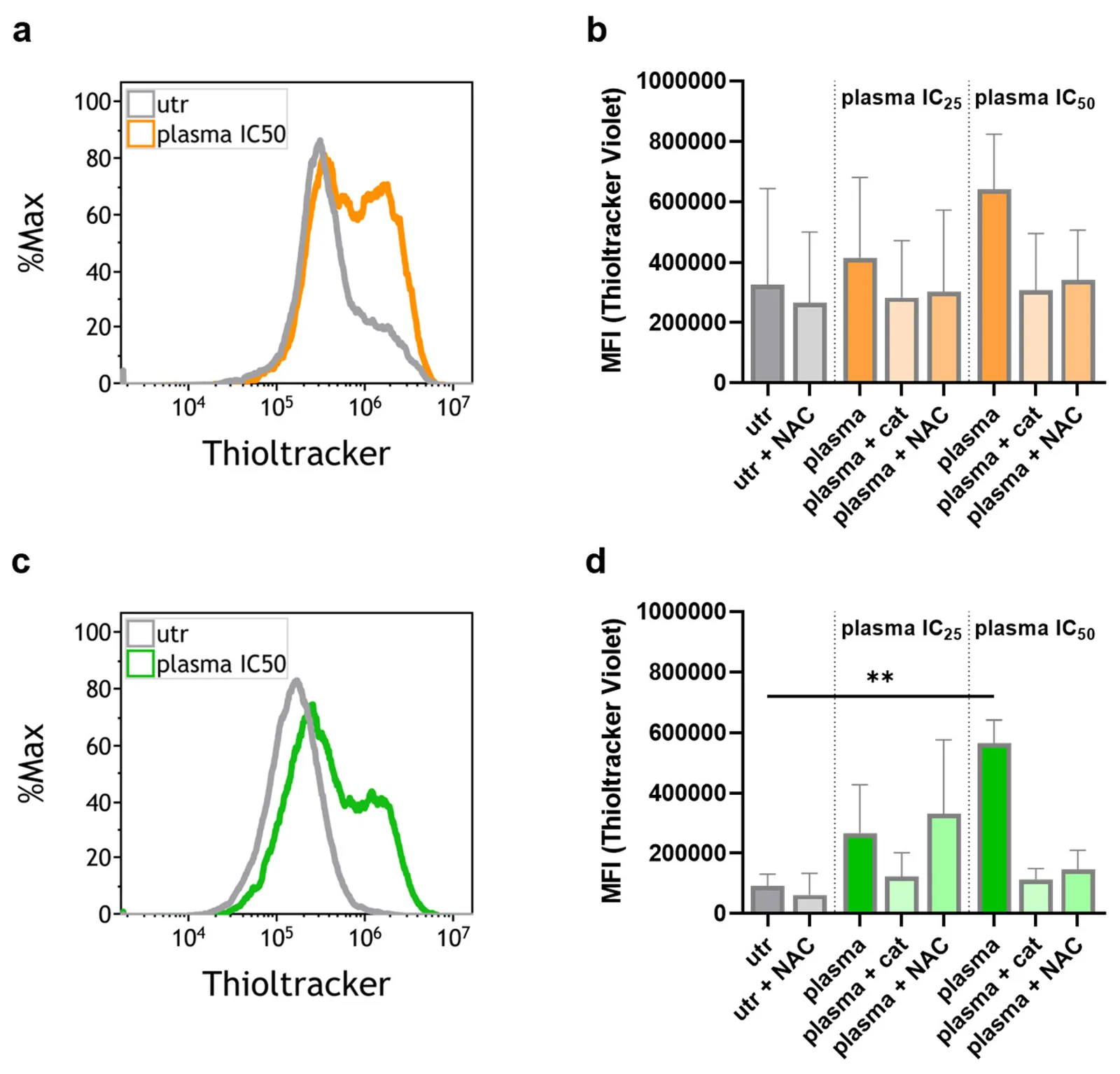
**Thioltracker analysis.** Flow cytometric analysis of cellular thiol levels in (**a**,**b**) HT-29 and (**c**,**d**) MUG-CCArly cells following medical plasma treatment and antioxidant scavenging. Mean fluorescence intensities (MFI) are shown for untreated and N-acetylcysteine (NAC) only controls, and cells treated with medical plasma for 30 s or 60 s, either alone or in the presence of 8 mM NAC or 5 µg/mL catalase (cat). Thioltracker fluorescence was measured after 2 h by flow cytometry to assess changes in cellular thiol levels in response to the different treatments. ** denotes statistically significant difference between groups (*p* < 0.01).

Both treatment groups differed significantly from the untreated control. At the same time, the difference between IC25 and IC25+NAC treatment was also significant (Figure 6b). The density of DAPI-positive cells increased significantly from 0.54 DAPI+/mm^2^ in untreated cells to 15.9 DAPI+/mm^2^ following IC25 treatment. In contrast, NAC pre-treatment reduced this response to 0.9 DAPI^+^/mm^2^ (Figure 6c). A marked increase in caspase-positive events was also observed, from 1.19 Caspase^+^/mm^2^ in untreated cells to 21.3 Caspase^+^/mm^2^ following IC_25_ treatment, which was significant compared to the untreated control. NAC pre-treatment reduced the number of caspase-positive cells to 7.76 Caspase^+^/mm^2^ (Figure 6d). Furthermore, MitoTracker fluorescence (Figure 6e) increased significantly from 5410 RFU in untreated cells to 11,744 RFU following IC_25_ treatment. Pre-treatment with NAC reduced fluorescence intensity towards control levels (Figure 6f). As in HT-29 cells, MitoSOX and CellROX fluorescence exhibited only minor treatment-dependent changes and did not differ significantly between groups (Figure 6g,h). To further investigate plasma-induced changes in cellular redox status, intracellular thiol levels were assessed 2h post-treatment using Thioltracker and flow cytometry in HT-29 and MUG-CCArly cells. Plasma treatment increased Thioltracker fluorescence in both cell lines. This increase was reduced by the addition of either NAC or catalase. These results are presented in the Appendix A (Figure A1).

### 3.4. Flow Cytometric Analysis Reveals Plasma-Induced Modulation of Stress- and Immune-Related Surface Markers

**Figure 7:**
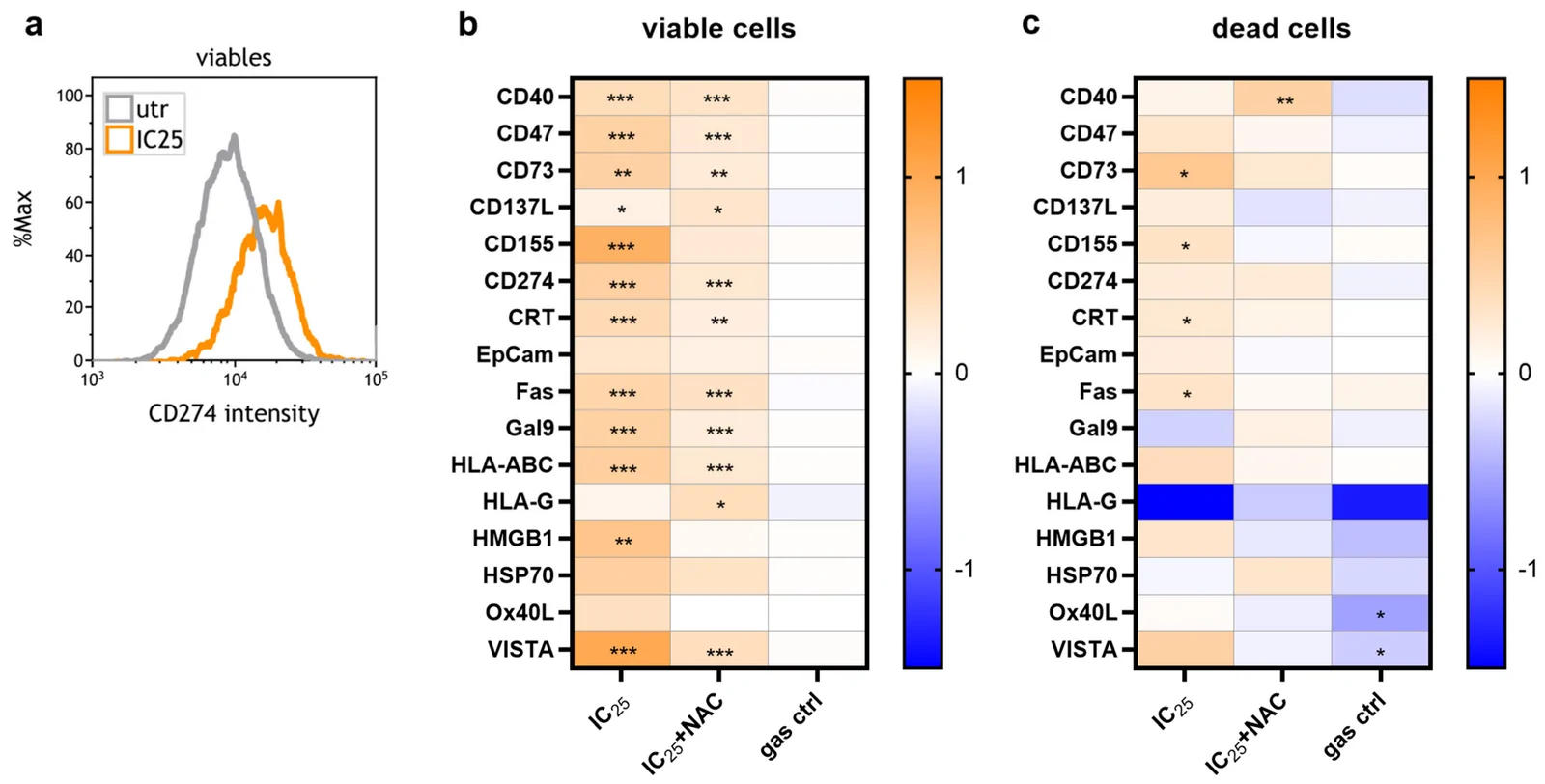
**Flow cytometric profiling of 16 stress- and immune-related surface marker expression in HT-29 cells.** Representative histogram of CD274 expression quantified in viable untreated control and IC_25_-treated HT-29 cells using flow cytometry (**a**); heatmaps depicting the expression profiles in the viable (**b**) and dead cell (**c**) populations 24 h after treatment. Data represent transformed fold changes relative to the untreated control (=0). Statistical significance relative to the untreated control was assessed using a one-sample *t*-test against a hypothetical mean of 0. Data are derived from four independent biological replicates, each analyzed in duplicate. Only statistically significant differences are marked with asterisks (* *p* < 0.05, ** *p* < 0.01, *** *p* < 0.001), whereas all other comparisons were not significant.

**Figure 8:**
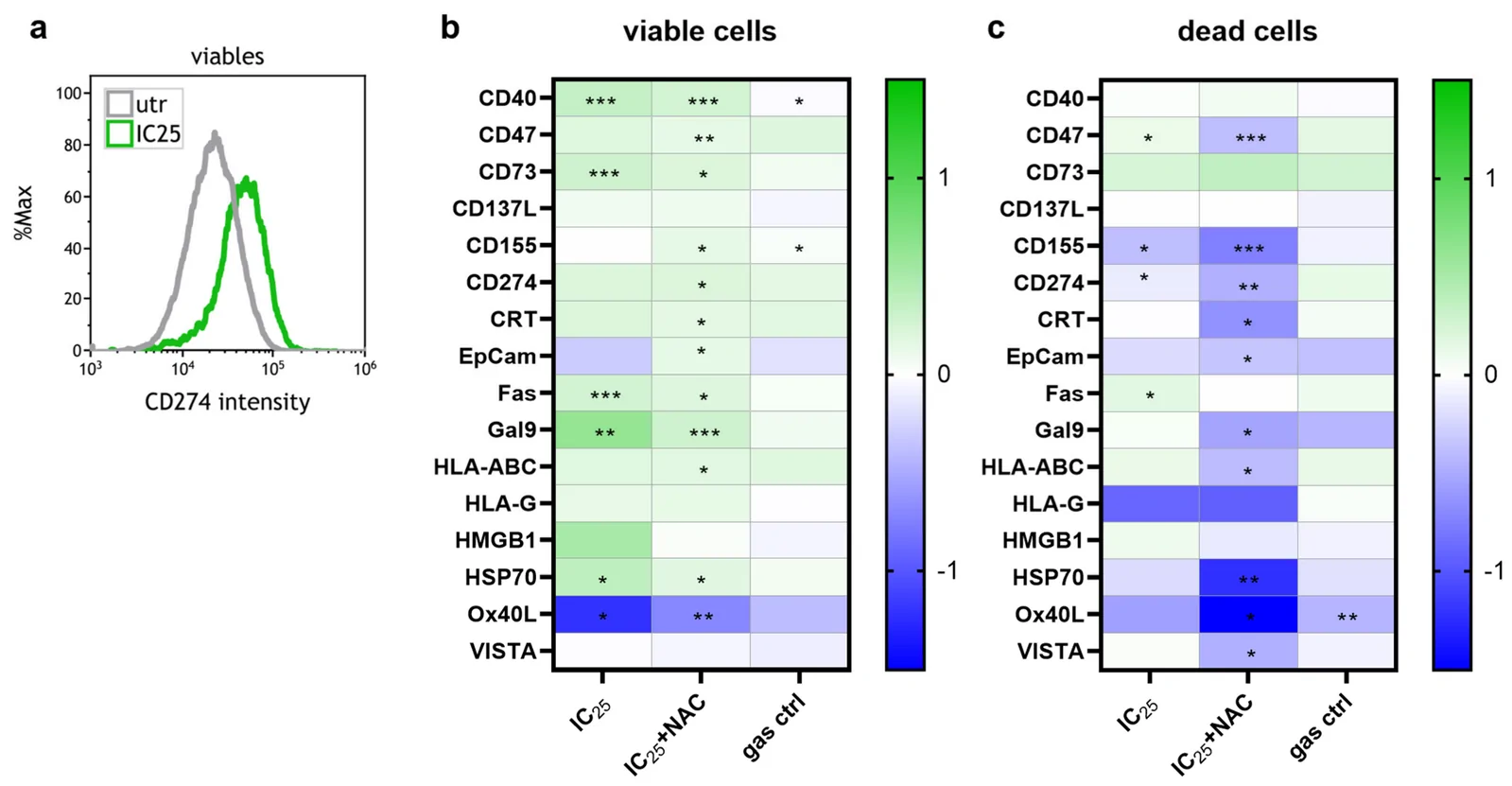
**Flow cytometric profiling of 16 stress- and immune-related surface marker expression in MUG-CCArly cells.** Representative histogram of CD274 expression quantified in viable untreated control and IC_25_-treated MUG-CCArly cells using flow cytometry (**a**); heatmaps depicting the expression profiles in the viable (**b**) and dead cell (**c**) populations 24 h after treatment. Data are transformed fold changes relative to the untreated control (=0). Statistical significance relative to the untreated control was assessed using a one-sample *t*-test against a hypothetical mean of 0. Data are derived from four independent biological replicates, each analyzed in duplicate. Only statistically significant differences are marked with asterisks (* *p* < 0.05, ** *p* < 0.01, *** *p* < 0.001), whereas all other comparisons were not significant.

In both cell lines HT-29 (Figure 7) and MUG-CCArly (Figure 8), flow cytometric quantification of the expression of 16 stress- and immune-related surface markers was performed 24 h after plasma treatment (Figure 7a and Figure 8a). Marker expression was normalized to untreated controls and analyzed separately in viable and dead cells. In viable HT-29 cells (Figure 7b), IC_25_ treatment significantly increased the surface expression of CD40, CD47, CD73, CD137L, CD155, CD274, CRT, Fas, Gal9, HLA-ABC, HMGB1 and VISTA. Except for CD137L, these effects were partially attenuated by pre-treatment with the RONS scavenger NAC. Interestingly, HLA-G was upregulated in the IC_25_+NAC group, although no significant change was observed after IC_25_ treatment alone. In the dead-cell fraction (Figure 7c), IC_25_ treatment increased the expression of CD73, CD155, CRT, and Fas. Again, pre-treatment with NAC largely attenuated these upregulations. For CD40, significant upregulation was detected exclusively in the IC_25_+NAC group, whereas OX40L and VISTA were significantly reduced only in the gas control group, despite the absence of significant modulation following IC_25_ treatment alone. In viable MUG-CCArly cells (Figure 8b), IC_25_ treatment significantly increased the expression of CD40, CD73, Fas, Gal9, and HSP70, whereas OX40L expression was significantly reduced. NAC partially restored the expression of these markers toward basal levels. Notably, viable MUG-CCArly cells also showed a significant upregulation of CD47, CD155, CD274, CRT, and HLA-ABC in the IC_25_+NAC group, although the upregulations observed in the IC_25_ group alone were not significant. A minor but significant increase in surface expression of CD40 and CD155 following gas control treatment was also observed. In dead MUG-CCArly cells (Figure 8c), IC_25_ treatment significantly increased the expression of CD47 and Fas, whereas CD155 expression was significantly reduced. Similar to the viable cells, several significant differences were observed exclusively in the IC_25_+NAC group. Notably, these markers, including CD47, DC155, CD274, CRT, EpCam, Gal9, HLA-ABC, HSP70, OX40L, and VISTA were all downregulated. Taken together, CD40, CD73, Fas, and Gal9 were consistently upregulated in viable cells of both cell lines after IC_25_ treatment, whereas most other responses were cell line- and viability-state-specific.

### 3.5. Gas Plasma Treatment Modulates Cytokine/Chemokine Secretion in GI Cancer Cells

**Figure 9:**
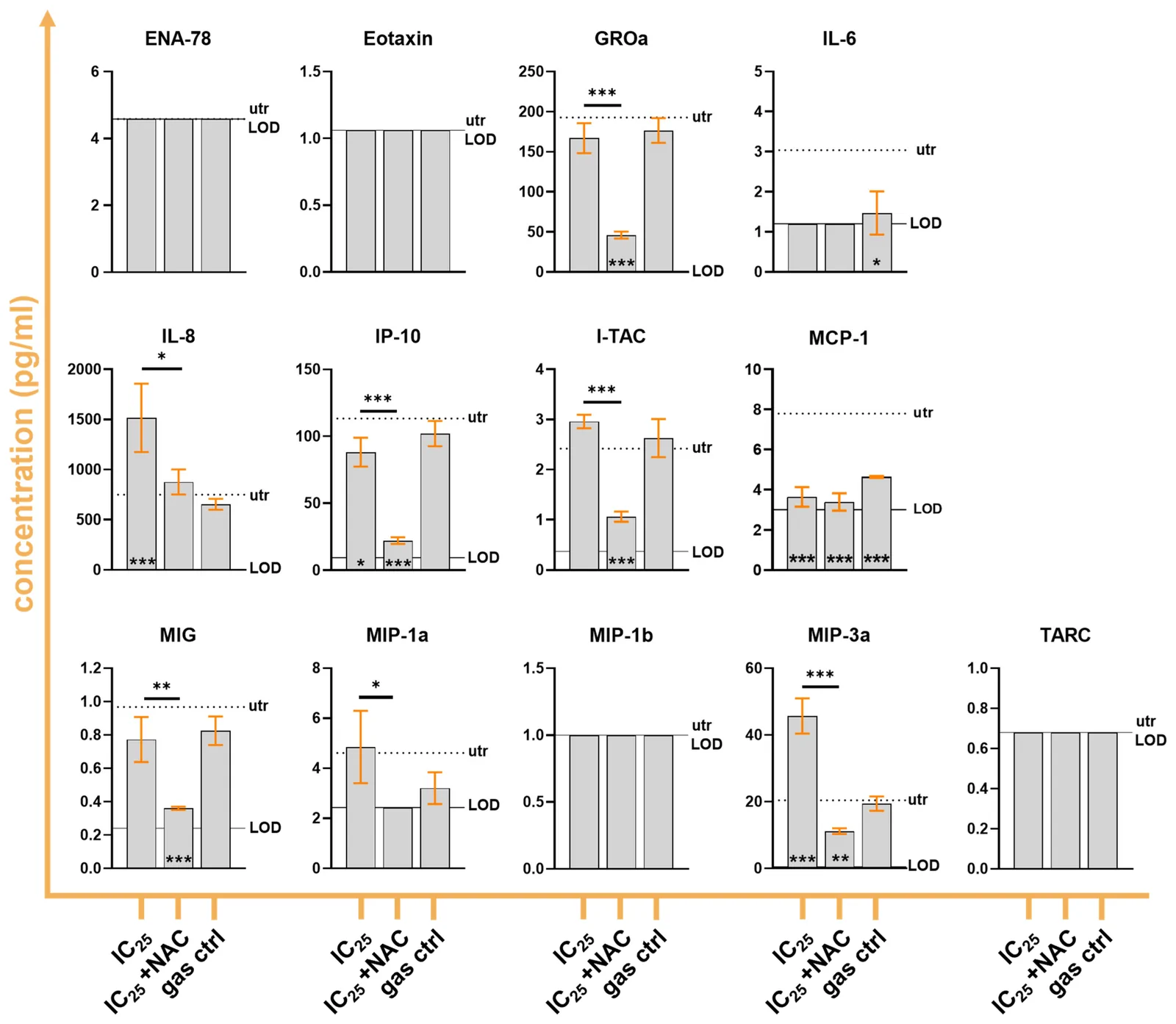
**Multiplex secretion profiling of HT-29 cells.** Quantification of the secretion of 13 cytokines and chemokines 24 h after treatment of HT-29 cells. The dashed lines indicate the untreated control, whereas the solid lines mark the limit of detection (LOD) for each analyte. Statistical significance relative to the untreated control was determined using ordinary one-way ANOVA followed by Dunnett’s multiple comparisons test and is indicated by asterisks within the corresponding bars. Differences between IC_25_ and IC_25_+NAC were assessed using an unpaired *t*-test with Welch’s correction applied in cases of significant variance differences (significant F-test result). Data are derived from four technical replicates. Only statistically significant differences are marked with asterisks (* *p* < 0.05, ** *p* < 0.01, *** *p* < 0.001), whereas all other comparisons were not significant.

**Figure 10:**
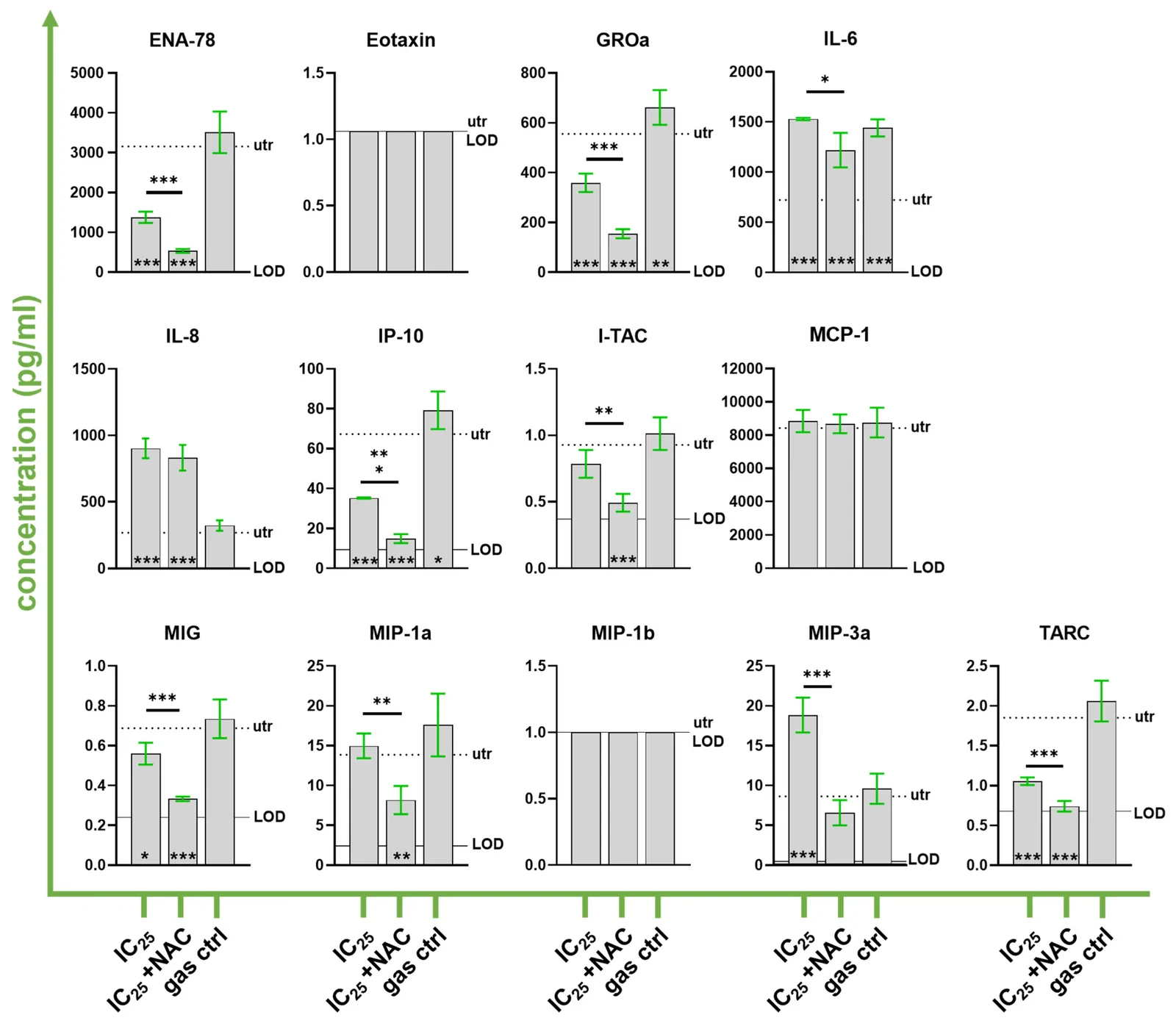
**Multiplex secretion profiling of MUG-CCArly cells.** Quantification of the secretion of 13 cytokines and chemokines 24 h after treatment of MUG-CCArly cells. The dashed lines indicate the untreated control, whereas the solid lines mark the limit of detection (LOD) for each analyte. Statistical significance relative to the untreated control was determined using ordinary one-way ANOVA followed by Dunnett’s multiple comparisons test and is indicated by asterisks within the corresponding bars. Differences between IC_25_ and IC_25_+NAC were assessed using an unpaired *t*-test with Welch’s correction applied in cases of significant variance differences (significant F-test result). Data are derived from four technical replicates. Only statistically significant differences are marked (* *p* < 0.05, ** *p* < 0.01, *** *p* < 0.001), whereas all other comparisons were not significant.

To investigate the impact of gas plasma treatment on the secretory phenotype of gastrointestinal cancer cells, cytokine concentrations were quantified in cell culture supernatants collected 24 h after exposure to the respective IC_25_ plasma dose. In HT-29 cells (Figure 9), plasma treatment induced a selective modulation of cytokine secretion. Among the 13 analytes assessed, IL-8 and MIP-3a were significantly increased in the IC_25_ group compared with untreated controls. In both cases, NAC supplementation significantly reduced cytokine concentrations relative to plasma treatment alone, resulting in levels closer to those observed in untreated cells. In contrast, IP-10 and MCP-1 concentrations were significantly decreased following plasma treatment. For IP-10, NAC further reduced cytokine levels towards the limit of detection, whereas the gas control exhibited concentrations comparable to the IC_25_ condition. MCP-1 concentrations were similarly reduced in the IC_25_, IC_25_+NAC, and gas control groups, all of which differed significantly from untreated controls. GROa, IL-6, I-TAC, MIG, and MIP-1a did not differ significantly between plasma-treated and untreated HT-29 cells; however, cytokine concentrations were significantly lower in the IC_25_+NAC group than in the IC_25_ condition. Concentrations of ENA-78, Eotaxin, MIP-1b, and TARC remained below the limit of detection in all treatment groups. A comparable but more pronounced response pattern was observed in MUG-CCArly cells (Figure 10). Similar to HT-29 cells, plasma treatment significantly increased IL-8 and MIP-3a secretion compared with untreated controls. For MIP-3a, NAC significantly attenuated this increase, whereas the reduction observed for IL-8 did not reach statistical significance. A general increase in IL-6 was detected for all treatment conditions but was significantly lower in the NAC group compared to the IC_25_-only group. In contrast, ENA-78, GROa, IP-10, MIG, and TARC were significantly decreased following plasma treatment and were further significantly reduced upon NAC supplementation. MCP-1 concentrations remained largely unchanged across all experimental groups. I-TAC and MIP-1a did not differ significantly between plasma-treated and untreated cells; however, both cytokines were significantly reduced in the IC_25_+NAC group compared with the IC_25_ condition. Concentrations of Eotaxin and MIP-1b remained below the limit of detection in all groups. Overall, plasma treatment induced cell line-specific alterations in cytokine secretion. The most consistent response across both cell lines was the increased release of IL-8 and MIP-3α following plasma exposure, whereas several cytokines displayed reduced concentrations, particularly in MUG-CCArly cells. NAC supplementation attenuated a subset of these responses.

## 4. Discussion

Gastrointestinal cancers remain among the most frequent types of cancer worldwide that require the development of novel therapy strategies. Medical plasma is emerging as a promising tool to expand the opportunities for anticancer treatments. This study aimed to investigate how the clinically approved argon plasma jet kINPen might confer anti-tumor effects in two gastrointestinal cancer cell lines. To this end, we investigated how the plasma source affected direct tumor cell death and immunomodulation through the production of reactive species.

The time-dependent changes in RONS levels confirmed that treatment of PBS with the argon-driven kINPen plasma generates both short- and long-lived reactive species in the liquid phase. The observed transient increase in APF and HPF fluorescence followed by a gradual decrease at longer treatment times is consistent with the transient formation and subsequent decay or secondary consumption of highly reactive species in the plasma-treated liquid [29,30]. In contrast, the continuous accumulation of H_2_O_2_ and nitrite reflects the formation of longer-lived RONS that can persist and accumulate during plasma exposure. Similar treatment-time-dependent formation of H_2_O_2_ and NO_2_^−^ has previously been reported for plasma-treated aqueous solutions, including experiments performed with the kINPen [9,31,32]. Thus, the present experiments do not represent a fundamentally new observation of RONS formation but rather characterize the temporal RONS profile generated under the specific plasma treatment conditions used in this study. At the subsequently selected treatment duration of approximately 30 s, both transient and long-lived RONS were already detectable, indicating that the cellular experiments were performed under conditions in which plasma-derived reactive species had been established in the liquid phase.

We observed a dose-dependent reduction in metabolic activity in both HT-29 and MUG-CCArly cells, demonstrating that plasma exposure exerts a clear cytotoxic effect on these gastrointestinal cancer cell lines. This is consistent with previous studies demonstrating that cold physical plasma and plasma-treated liquids can induce concentration- or plasma treatment-time-dependent cytotoxicity in cancer cells [5]. The sigmoidal dose–response relationships and comparable IC_25_ values further indicated that both cell lines respond within a similar treatment-time range. Given the plasma treatment-time-dependent generation of RONS observed in PBS, the subsequent experiments were therefore aimed at determining whether the cytotoxic effects observed at this plasma treatment duration are related to plasma-derived RONS and, if so, which reactive species or mechanisms contribute to the cellular response.

Plasma exposure induced cellular alterations visible in the reduction in cell-covered area, reflecting decreased cell proliferation, and an increase in the proportion of DAPI-positive cells, indicating a higher fraction of dead cells. The increase in caspase-positive cells further supports the induction of apoptotic cell death that was already reported for plasma treatment in various cancer entities, including tumor tissue [33,34,35,36]. In addition to the observed activation of the executioner caspases 3 and 7, which are well-known players in apoptosis [37,38], the loss of mitochondrial membrane potential belongs to the central events in the early phase of the apoptotic pathway [39]. The observed increase in MitoTracker fluorescence was therefore unexpected and may reflect additional changes in mitochondrial content or cellular properties following plasma exposure. Since MitoTracker fluorescence is influenced by mitochondrial membrane potential and may also reflect changes in mitochondrial content, the increased signal cannot be directly interpreted as evidence of increased mitochondrial abundance. Although alterations in mitochondrial mass associated with cell cycle arrest could potentially contribute to the observed increase in fluorescence [40], this possibility remains speculative, as mitochondrial mass and membrane potential were not independently assessed in the present study. The increase in DAPI- and Caspase-positive cells was largely attenuated or completely prevented by the RONS scavenger N-acetylcysteine (NAC), supporting the involvement of redox-dependent processes in the plasma-induced cellular response. However, as NAC is not specific for individual reactive species but rather broadly detoxifies by direct scavenging of various RONS (e.g., HO•, H_2_O_2_) and indirectly by restoring the intracellular glutathione (GSH) levels [41], these findings support a contribution of RONS without identifying the specific species responsible. Overall, the response was more pronounced in MUG-CCArly cells, where all observed changes reached statistical significance. In contrast, none of the corresponding effects in HT-29 cells were significant despite clear differences in mean values. CellROX, which provides a measure of cellular oxidative stress, and MitoSOX, which preferentially detects mitochondrial superoxide, showed only minor and non-significant changes in either cell line. Thus, no pronounced increase in cellular oxidative stress or mitochondrial superoxide-associated fluorescence was detectable immediately after plasma treatment. Nevertheless, the strong attenuation of the cytotoxic phenotype by NAC supports the involvement of redox-dependent processes. It provides a basis for the subsequent experiments investigating the causal contribution of plasma-derived RONS.

The surface marker analysis extends the previous findings by showing that plasma exposure at the IC_25_ condition also altered the surface phenotype of both gastrointestinal cancer cell lines 24 h after plasma treatment. In viable cells, CD40, CD73, Fas, and Gal9 were consistently upregulated in both HT-29 and MUG-CCArly cells, suggesting that the plasma-induced stress response is accompanied by reproducible changes in immune- and stress-related signaling. The increase in Fas is consistent with the apoptotic response observed by high-content imaging and may indicate an increased susceptibility to Fas-mediated apoptotic signaling [42]. CD40 upregulation may reflect activation of immune-regulatory pathways and may also contribute to enhanced tumor immunogenicity and, upon CD40 engagement, to tumor-cell growth inhibition or apoptosis [43]. In contrast, increased CD73 may represent a counter-regulatory response, as this ectonucleotidase converts extracellular AMP to immunosuppressive adenosine and can therefore contribute to an immunosuppressive tumor microenvironment [44,45]. Similarly, Gal9 is a context-dependent immunoregulatory molecule that can modulate T-cell responses through interactions with receptors including TIM-3 [46]. The increased expression of CRT and HMGB1 in viable HT-29 cells is particularly relevant in the context of immunogenic cell death, as CRT exposure and HMGB1 release are established hallmarks of immunogenic stress and have already been described following plasma treatment of cancer cells [47,48]. At the same time, several markers associated with immune regulation or immune evasion, including CD47, CD73, CD155, CD274, Gal9, and VISTA, were altered following plasma treatment, particularly significant in HT-29 cells. These changes indicate that plasma-induced cellular stress is accompanied not only by potentially immunostimulatory signals but also by modulation of pathways that can regulate immune recognition. The increase in CD47 is of particular interest because CD47 functions as a "don’t-eat-me" signal and has previously been reported to decrease following plasma treatment in a cancer model [48]. Moreover, plasma-induced oxidation has been shown to alter the conformation of CD47 as an early RONS-mediated effect [49]. Notably, CD47 levels returned towards baseline within 24 h in that study, suggesting that this initial plasma-induced modification is only transient. Thus, the increased CD47 signal observed in the present study at 24 h may not reflect a direct effect of plasma on CD47, but rather a subsequent cellular response to plasma-induced stress. An increase in the immune checkpoint CD274 (PD-L1) upon plasma treatment is also in line with previous plasma studies [50,51], and reactive oxygen species in general seem to induce PD-L1 expression frequently [52]. Although the intrinsic upregulation of PD-L1 in tumor cells is known to be beneficial for immunoevasion, it is assumed that boosting this upregulation might even augment the sensitivity of cancer cells towards immune checkpoint inhibition (anti-PD-L1 blockade) [53]. The strong attenuation of many plasma-induced changes by NAC further supports an important contribution of redox-dependent mechanisms to the observed phenotypic alterations. This is consistent with the established role of plasma-derived RONS in regulating intracellular signaling, stress responses, and cell death. However, the occurrence of significant changes exclusively in the IC_25_+NAC group should be interpreted with caution. In most cases, particularly in the viable MUG-CCArly population, the magnitude of these changes was comparable to that observed following IC_25_ plasma treatment alone. However, they did not reach statistical significance. Moreover, the observation of significant increases in these markers following IC_25_ plasma treatment in HT-29 cells supports a predominantly plasma-driven response rather than a NAC-specific induction. Thus, the data do not provide clear evidence that NAC directly induces the observed marker changes, but rather suggest that modulation of the cellular redox state may influence the magnitude or detectability of plasma-induced responses. The significantly reduced marker expression in the IC_25_+NAC group observed in the dead MUG-CCArly cell population may additionally reflect differences in the composition or state of the dying cell population following NAC pre-treatment, although this interpretation remains speculative. Overall, the observed surface phenotype is consistent with plasma-induced immunogenic stress but also reflects concurrent immunoregulatory changes. Thus, plasma treatment appears to induce a complex stress-adaptive response rather than a uniformly immunogenic phenotype. In future studies, cocultures of plasma-treated cancer cells and immune cells could provide more precise insights into immune regulatory features of the plasma treatment.

The multiplex analysis further demonstrated that plasma treatment induces a selective and cell line-dependent remodeling of the secretory phenotype. The most consistent response across both cell lines was the increased secretion of IL-8 and MIP-3a/CCL20 following plasma exposure. Both chemokines are involved in inflammatory signaling and immune-cell recruitment. However, IL-8 may also promote tumor progression through CXCR1/2 signaling, highlighting the potentially context-dependent effects of its increased secretion [54,55]. Similarly, elevated MIP-3a/CCL20 levels may also contribute to a tumor-promoting microenvironment by recruiting CCR6-expressing immune cells [56]. In general, the cytokine profile did not indicate a uniformly pro-inflammatory or immunostimulatory response. Overall, plasma treatment appears to selectively enhance some immunomodulatory signals while suppressing others, consistent with previous reports describing heterogeneous cytokine responses following plasma exposure [57,58]. This mixed pattern is consistent with the simultaneous modulation of potentially immunostimulatory and immunoregulatory surface markers observed in the flow cytometric analysis. NAC attenuated several of the plasma-associated changes, particularly the increase in MIP-3a, supporting a contribution of redox-dependent mechanisms to the altered secretory phenotype. However, NAC did not simply restore cytokine concentrations towards untreated levels. Instead, several cytokines, including GROa, IP-10, I-TAC, MIG, MIP-1a, and MIP-3a, were further reduced following NAC supplementation. This finding suggests that NAC modifies the cellular response on its own rather than simply reversing plasma-induced cytokine alterations. Given that NAC can modulate intracellular redox and glutathione homeostasis, its effects may extend beyond the neutralization of reactive species and involve downstream stress-responsive and redox-sensitive signaling pathways [59]. Overall, the data support a cell line-dependent, redox-sensitive, and mixed immunomodulatory response rather than a simple pro-inflammatory or immunogenic effect. Importantly, altered cytokine concentrations may also partly reflect the reduced number or altered state of viable cells following plasma exposure. Therefore, the present findings demonstrate changes in the secretory environment but do not by themselves establish a functional enhancement or suppression of anti-tumor immunity. More complex experiments investigating the direct effects or interactions of the plasma-treated GI cancer cells on or with immune-relevant cells would be needed to determine whether it is more of a pro- or anti-inflammatory response.

One limitation of the present study is that the experimental design primarily addressed the effects of direct medical plasma exposure and did not allow a comprehensive dissection of the individual contributions of the diverse RONS generated by the plasma. Although the observed cytotoxic effects were associated with oxidative stress, the derived biological responses should not be attributed exclusively to RONS. The analytical and biological methods used in this study do not allow an unambiguous differentiation between the effects of individual ROS and RNS species, their secondary reaction products, and plasma-induced changes in the surrounding cell-culture environment. Therefore, the present data should be interpreted as evidence for a broader redox-associated response rather than as proof of a solely RONS-mediated mechanism. Moreover, the current experimental setup does not distinguish between effects elicited by short-lived species acting during direct plasma treatment and those mediated by longer-lived RONS or secondary products that persist in the culture medium after treatment. This distinction is particularly relevant because plasma-treated media, including liquids and hydrogels, can preserve and transfer longer-lived reactive species to biological targets, thereby enabling delayed, sustained, and potentially localized biological effects [30]. Such delivery-based approaches may partly overcome the limited lifetime and penetration depth of short-lived species produced during direct plasma exposure. For example, recent work has demonstrated that plasma-generated RONS can be transferred via plasma-treated liquid into cryo-microneedle patches, resulting in therapeutic effects in a melanoma [60]. While our findings were obtained following direct treatment with the kINPen, indirect RONS-mediated effects through reactive species retained in the culture medium cannot be excluded.

## 5. Conclusions

The present findings demonstrate that medical argon plasma induces a multifaceted response in gastrointestinal cancer cells, encompassing cytotoxicity, alterations in cell-surface phenotype and modulation of the secretory cytokine profile. The observed effects were partly redox-dependent but also varied between cell lines and were not uniformly immunostimulatory. Thus, plasma treatment appears to induce a complex cellular stress response with both potentially immunostimulatory and immunoregulatory features. These findings support further investigation of medical plasma as a multimodal therapeutic approach in gastrointestinal oncology, particularly with regard to its effects on the tumor-immune microenvironment.

## Figures and Tables

**Table 1:** Monoclonal antibodies used for flow cytometric analysis of surface marker expression.

Ligand	Fluorochrome	Clone
CD40	FITC	5C3
CD47	BV605	CC2C6
CD73	BV421	AD2
CD137L	PC7	5F4
CD155	APC	SKII.4
CD274	BV650	29E2A3
CRT	PE	FMC 75
EpCam	BV605	9C4
Fas	BV510	DX2
Gal9	PerCP-Cy5.5	9M1-3
HLA-ABC	BV510	W6/32
HLA-G	PC7	87G
HMGB1	PerCP-Cy5.5	B5C8
HSP70	APC	N27F3-4
Ox40L	PE/Dazzle 594	RM134L
VISTA	APC/Cy7	730823

## Data Availability

The underlying data of this manuscript are available from the corresponding author upon reasonable request.
